# Joint Response to Exercise Is Affected by Knee Osteoarthritis: An Infrared Thermography Analysis

**DOI:** 10.3390/jcm12103399

**Published:** 2023-05-11

**Authors:** Luca De Marziani, Angelo Boffa, Simone Orazi, Luca Andriolo, Alessandro Di Martino, Stefano Zaffagnini, Giuseppe Filardo

**Affiliations:** 1 Clinica Ortopedica e Traumatologica 2, IRCCS Istituto Ortopedico Rizzoli, 40136 Bologna, Italy; 2 Applied and Translational Research (ATR) Center, IRCCS Istituto Ortopedico Rizzoli, 40136 Bologna, Italy

**Keywords:** infrared thermography, knee, osteoarthritis, joint temperature, inflammation, response, exercise

## Abstract

Infrared thermography can be used to evaluate the inflammation characterizing the joint environment of OA knees, but there is limited evidence on the response to physical exercise. Identifying the response to exercise of OA knees and the influencing variables could provide important information to better profile patients with different knee OA patterns. Sixty consecutive patients (38 men/22 women, 61.4 ± 9.2 years) with symptomatic knee OA were enrolled. Patients were evaluated with a standardized protocol using a thermographic camera (FLIR-T1020) positioned at 1 m with image acquisition of an anterior view at baseline, immediately after, and at 5 min after a 2-min knee flexion–extension exercise with a 2 kg anklet. Patients’ demographic and clinical characteristics were documented and correlated with the thermographic changes. This study demonstrated that the temperature response to exercise in symptomatic knee OA was affected by some demographic and clinical characteristics of the assessed patients. Patients with a poor clinical knee status presented with a lower response to exercise, and women showed a greater temperature decrease than men. Not all evaluated ROIs showed the same trend, which underlines the need to specifically study the different joint subareas to identify the inflammatory component and joint response while investigating knee OA patterns.

## 1. Introduction

Infrared thermography is an established method that is able to detect the infrared radiation emitted by the human body, which correlates with the temperature distribution of a defined region [1]. This technology, used for the first time in the 1960s [2], allows us to identify and locate thermal abnormalities characterized by an increase or decrease in temperature at the skin surface, which can reflect the status of a specific pathology [3]. In particular, infrared thermography has been proposed as a method to evaluate conditions with an inflammatory component, which plays a central role in the pathophysiology of several diseases [4]. The awareness of the role of inflammation within a wide range of diseases as well as the technological advancements in cameras and improvements in software used for image analysis has led to increased use of infrared thermography in different scientific fields, from dermatology to oncology [5,6]. Recently, the use of infrared thermography has been proposed in the orthopedic field as a potential method for evaluating patients with knee osteoarthritis (OA) to better characterize the pathology and guide personalized treatment [4].

Knee OA is one of the most common musculoskeletal diseases. It is characterized by the deterioration and loss of articular cartilage with concomitant structural and functional changes across the entire joint [7]. Inflammation plays a key role in the pathophysiology of knee OA, with the involvement of the synovial membrane and the release of several proinflammatory cytokines [8,9,10]. The inflammatory component leads to an increase in blood flow that can manifest clinically as redness and heat as well as joint swelling and pain [4]. Infrared thermography has been proven, through the evaluation of the skin temperature of the knee, to be able to evaluate the inflammation component that characterizes the joint environment of OA knees [11,12,13,14]. However, although the use of infrared thermography in this setting is growing, there is limited evidence on its use for the evaluation of the inflammatory response to physical exercise in OA knees. While a few reports suggest that there are activity-related changes in OA knees, despite documenting temperature-induced changes after exercise, no studies have evaluated possible factors influencing the temperature response [15,16]. Identifying the response to exercise of OA knees and the variables that influence this response could provide important information to better profile patients with different knee OA patterns.

The aim of this study was to evaluate, through infrared thermography, the response to a knee flexion–extension exercise and identify the clinical and demographic variables able to influence this response in patients with symptomatic knee OA.

## 2. Materials and Methods

This study was approved by the hospital ethics committee of the IRCCS Istituto Ortopedico Rizzoli, Italy (n. 0017413). Patient screening was performed by orthopedic physicians in a research outpatient department of a highly specialized referral center for orthopedics focused on patients with knee OA. The evaluation was performed from December 2021 to December 2022. Informed consent was obtained from each patient prior to study participation. Patients were clinically evaluated for their eligibility for study inclusion according to the following criteria: patients with monolateral symptomatic knee OA (Kellgren–Lawrence grade ≥ 2) with a history of chronic pain or swelling (for at least 6 months) were included in the study. The exclusion criteria were as follows: previous total knee arthroplasty; history of trauma or intra-articular injections within 6 months before treatment or knee surgery within 12 months; the presence of concomitant lesions causing knee pain or swelling, including radiculopathy; clinical signs of dermatological and vascular conditions; neoplasms; systemic disorders (i.e., uncontrolled diabetes); uncontrolled metabolic disorders of the thyroid; severe cardiovascular diseases; rheumatoid arthritis and other inflammatory arthropathies; hematological diseases; infections; immunodepression; anticoagulant or antiaggregant therapy; the use of nonsteroidal anti-inflammatories or other analgesic drugs in the 5 days before the evaluation. According to the Thermographic Imaging in Sports and Exercise Medicine (TISEM) guidelines [17] as well as considering the guidelines of the American Academy of Thermology [18], patients were asked to respect some instructions: avoidance of exercise and physical activity within 48 h; avoidance of alcohol beverages, smoking, caffeine, large meals, any type of ointment, cosmetics, and showering within 4 h; avoidance of ice or lotion applications within 48 h; and avoidance of knee exposure to the sun for long periods during the week prior to the examination.

A total of 60 consecutive patients with symptomatic knee OA were enrolled in accordance with the inclusion/exclusion criteria. Among them, 38 patients were men and 22 were women, aged 61.4 ± 9.2 years, and with a body mass index (BMI) of 25.4 ± 3.0. All demographic and clinical characteristics are reported in Table 1.

After enrollment, patients were evaluated clinically thorough knee-specific patient reported outcome measurements (PROMs), including the International Knee Documentation Committee (IKDC) subjective score, the Knee Injury and Osteoarthritis Outcome Score (KOOS) subscales, the Tegner score for activity level, the Visual Analogue Scale (VAS) for pain, and the PainDETECT questionnaire for the evaluation of the neuropathic pain component. Clinical questionnaires were administered via paper questionnaires during clinical visits in the research outpatient clinic. Patients completed the questionnaires, and doctors were available in case of questions. Moreover, the IKDC objective scores were evaluated by the clinician. All participants underwent weight-bearing antero-posterior radiographs to assess the baseline OA severity according to the Kellgren–Lawrence classification. Finally, the skin temperature of the knee affected by symptomatic OA was evaluated with thermographic imaging.

### 2.1. Infrared Thermography Procedure, Exercise, and Analysis

The infrared imaging evaluation was performed in a dedicated outpatient clinic shielded from direct sunlight and with the temperature controlled at 23.0 °C [19,20] and a mean humidity of 45 ± 3%. Image acquisition was performed between 14:00 and 17:00 to minimize the circadian temperature variations. According to Marins et al. [21], the thermalization period was 10 min. To speed up thermalization, patients were asked to remove trousers, shoes, and socks, remain seated and undressed on the lower limbs with light clothing (such as a t-shirt) on the top, and not touch their knees. The patient only rested the buttocks region on the medical bed, while the remaining parts of the lower limbs had no contact with other objects or body parts; only feet without socks touched a paper towel, thus separating them from direct contact with the floor. Thermograms were acquired using a FLIR T1020 thermographic camera (FLIR^®^ Systems, Stockholm, Sweden) with a resolution of 1024 × 768 pixels and a thermal sensitivity of 0.02 °C. The camera was positioned at a distance of 1 m, perpendicular to the knee and adjusted to the patellar height [22]. After the patient was acclimatized, he was positioned on a designated floor map, and image acquisition (T0) of an anterior view was performed using the autofocus mode.

**Figure 1:**
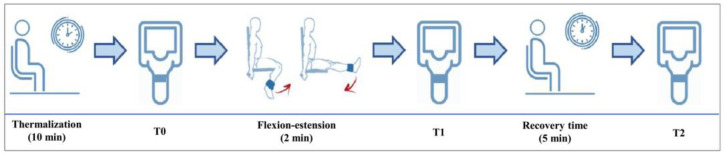
Timeline of the study.

Then, one 2 kg anklet was positioned on the ankle of the symptomatic lower limb of the patient. At this point, with the patient seated, a knee flexion–extension exercise was performed for 2 min at the rate of one extension every 2 s (1 s flexion phase and 1 s extension phase). A metronome was used to standardize pacing. Immediately after performing this exercise, the anklet was removed, and the patient was positioned again on the floor map and a second anterior view image was acquired (T1). Afterwards, the patient waited in the room for 5 min in a sitting position without touching or moving the lower limbs. At the end of this resting period, the patient was positioned on the floor map and a third anterior view image was acquired (T2). Finally, maintaining the same position of the knee, an anatomical marker (circular adhesive of 2 cm in diameter) was placed at the center of the patella to obtain a further image in the anterior view in order to facilitate the precise subsequent location of the patella in the analysis of the previous infrared images (Figure 1). 

During the image analysis process, the three anterior images acquired at T0, T1, and T2 were aligned side by side with the image with the patellar marker on the computer screen, and a template indicating the region of interests (ROIs) was centered over the patella of each unmarked image, using the marked image as a guide [23,24]. The ROIs were defined as follows: the patellar area was a square, 6 cm in diameter, divided into the medial patella and lateral patella (each area 6 cm high and 3 cm wide); the suprapatellar area was the area 3 cm over the patella; the medial and lateral areas were the regions 3 cm below the patella on its medial and lateral sides, respectively. The mean temperatures were extracted using ResearchIR software (FLIR^®^ Systems, Stockholm, Sweden) to determine the overall knee area and the 5 ROIs: medial patella, lateral patella, suprapatellar, and medial and lateral knees.

### 2.2. Statistical Analysis

All continuous data are expressed in terms of the mean and the standard deviation of the mean and range, and the categorical data are expressed as frequencies and percentages. The Shapiro–Wilk test was performed to test normality of continuous variables. The Levene test was used to assess the homoscedasticity of the data. The Repeated Measures General Linear Model (GLM) with the Sidak test for multiple comparisons was performed to assess the differences in different areas. The ANOVA test was performed to assess the between-group differences of continuous, normally distributed, and homoscedastic data; the Mann–Whitney nonparametric test was used otherwise. The ANOVA test, followed by the post-hoc Sidak test for pairwise comparisons, was performed to assess the among-group differences of continuous, normally distributed, and homoscedastic data, the Kruskal–Wallis nonparametric test, followed by the post-hoc Mann–Whitney test with Bonferroni correction for multiple comparisons, was used otherwise. The Spearman rank correlation was used to assess correlations between temperature and continuous data; the Kendall tau rank correlation was used for ordinal data. For all tests, *p* < 0.05 was considered significant. All statistical analyses were performed using SPSS v.19.0 (IBM Corp., Armonk, NY, USA).

## 3. Results

### 3.1. Temperature Changes

**Figure 2:**
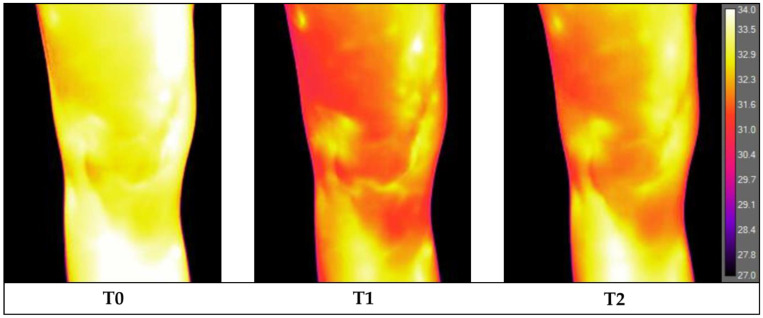
Thermographic basal image (**T0**), at the end of the 2-min flexion–extension exercise (**T1**) and after the 5-min rest period (**T2**).

The mean temperature of the total knee significantly changed after exercise, ranging from the baseline (T0) value of 32.13 ± 1.07 °C to 31.86 ± 1.12 °C at T1 and 31.94 ± 1.10 °C at T2 (*p* = 0.002). In detail, the mean temperature of the total knee detected at T0 was higher compared to that at T1 (*p* = 0.001) with a mean difference T0-T1 (∆T0 T1) of 0.27 °C and compared to that at T2 (*p* = 0.036) with a mean difference T0-T2 (∆T0 T2) of 0.20 °C. No significant differences in the mean temperature of the total knee were found between T1 and T2 (Figure 2).

**Figure 3:**
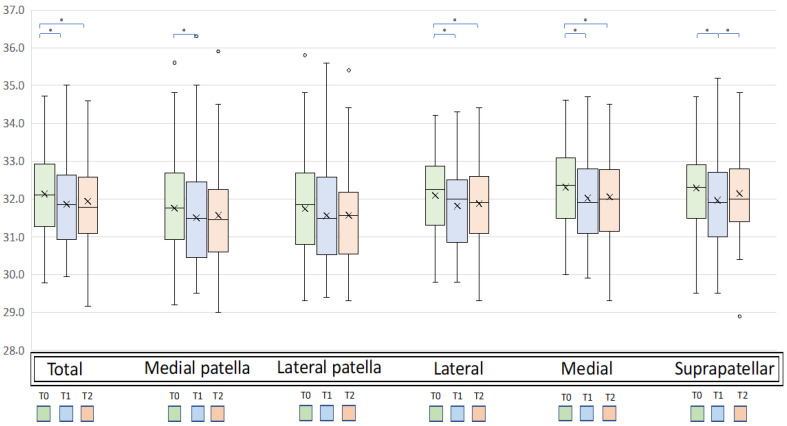
Mean temperatures of the total knee and subareas at T0, T1, and T2 (* *p* < 0.05, post-hoc Sidak test). Box-and-whisker plots showing median values and interquartile ranges. The “x” represents the mean temperature. T0: baseline; T1: immediately after performing this exercise; T2: 5 min after performing this exercise.

Similar changes in the mean temperature after exercise were observed for all knee subareas (Figure 3). In particular, statistically significant changes (ANOVA test) were detected for the lateral (*p* = 0.001), medial (*p* = 0.002), suprapatellar (*p* < 0.0005), and medial patella subareas (*p* = 0.011), while no significant changes were observed for the lateral patella subarea. A higher change in temperature from T0 to T1 (∆T0-T1) was found for the suprapatellar area (*p* < 0.0005) with a mean ∆T0-T1 of 0.33 °C, while a smaller change was detected for the lateral patella area (*p* = n.s) with a mean ∆T0 T1 of 0.19 °C. A higher change in temperature from T0 to T2 (∆T0 T2) was found for the medial area with a mean ∆T0-T2 of 0.25 °C, while a smaller change was detected for the suprapatellar area (n.s) with a mean ∆T0-T2 of 0.15 °C. Analyzing the changes in temperature between T1 and T2 (∆T1-T2) showed a significant increase in the suprapatellar area (+0.18, *p* = 0.030), while no differences were found in any other areas.

### 3.2. Influences of Demographic Variables

**Figure 4:**
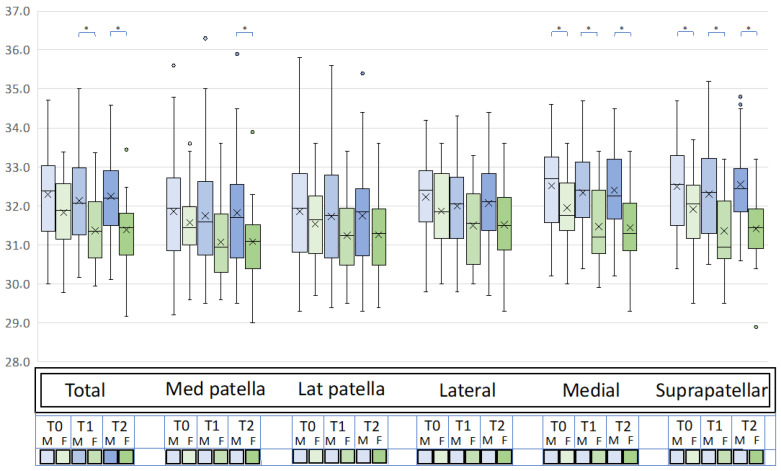
Differences in the total mean knee temperature and subareas at T0, T1, and T2 in men and women (* *p* < 0.05). Box-and-whisker plots showing median values and interquartile ranges. “x” represents the mean temperature. F: females; M: males; T0: baseline; T1: immediately after performing this exercise; T2: 5 min after performing this exercise.

Sex influenced the temperature changes after the exercise (Figure 4). Women had a greater decrease in temperature than men after exercise (∆T0-T1) in the total knee (−0.47 ± 0.64 vs. −0.16 ± 0.49, *p* = 0.021) and the medial (−0.48 ± 0.73 vs. −0.17 ± 0.50, *p* = 0.042) and suprapatellar (−0.56 ± 0.70 vs. −0.19 ± 0.55, *p* = 0.022) areas. Similarly, women had a greater decrease in temperature between T0 and T2 (∆T0-T2) in the total knee (−0.46 ± 0.49 vs. −0.05 ± 0.59, *p* = 0.009) and the medial (−0.52 ± 0.50 vs. −0.10 ± 0.60, *p* = 0.007), medial patella (−0.48 ± 0.62 vs. −0.03 ± 0.60, *p* = 0.018), and suprapatellar (−0.50 ± 0.51 vs. −0.06 ± 0.66, *p* = 0.001) areas. No significative differences were found in ∆T1T2 between women and men. The other demographic characteristics, including age, BMI, OA grade, sport activity level, smoking status, previous surgery, and symptom onset did not influence the thermic response after exercise.

### 3.3. Influence of Clinical Variables

**Figure 5:**
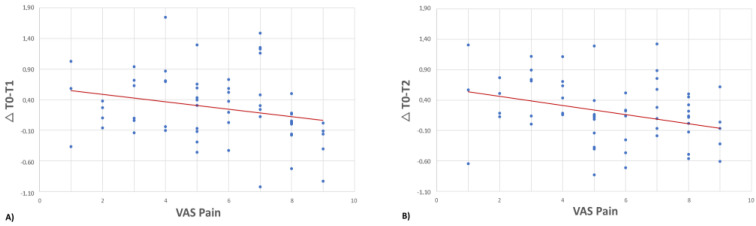
VAS pain is negatively correlated with ∆T0-T1 (**A**, rho = −0.296, *p* = 0.022) and ∆T0-T2 (**B**, rho = −0.318, *p* = 0.013).

The ∆T0-T1 of the total knee was negatively correlated with the VAS score (rho = −0.296, *p* = 0.022), with a higher temperature change occurring in patients with lower VAS scores (Figure 5a). This correlation was confirmed for the medial area (rho = −0.320, *p* = 0.013) and medial patella area (rho = −0.294, *p* = 0.023). The medial area was also positively correlated with the IKDC subjective score (rho = 0.363, *p* = 0.004) and the KOOS ADL subscale (rho = 0.259, *p* = 0.045), with higher temperature changes occurring in patients with better clinical values. 

The ∆T0-T2 of the total knee was negatively correlated with the VAS score (rho = −0.318, *p* = 0.013), with a higher temperature change occurring in patients with lower VAS scores (Figure 5b). Similar trends were observed for all subareas: lateral (rho = −0.256, *p* = 0.049), medial (rho = −0.365, *p* = 0.004), suprapatellar (rho = −0.270, *p* = 0.037), medial patellar (rho = −0.310, *p* = 0.016), and lateral patellar areas (rho = −0.271, *p* = 0.036). The IKDC subjective score was positively correlated with the ∆T0-T2 of the total knee (rho = 0.299, *p* = 0.020) and the medial area (rho = 0.371, *p* = 0.004), with higher temperature changes occurring in patients with higher IKDC subjective scores. The ∆T0-T2 of the total knee and the medial area were also positive correlated with the KOOS ADL subscale (rho = 0.256, *p* = 0.048 and rho = 0.298, *p* = 0.021, respectively) and the KOOS Sport/Rec subscale (rho = 0.307, *p* = 0.017 and rho = 0.369, *p* = 0.004, respectively), with higher temperature changes occurring in patients with higher activity levels. The PainDETECT questionnaire scores were negatively correlated with the ∆T0-T2 of the total knee (rho = −0.270 and *p* = 0.037) and the medial area (rho = −0.281, *p* = 0.030), with lower temperature variations occurring in patients with higher PainDETECT scores.

No correlations were found between ∆T1T2 temperatures of all areas and the clinical variables analyzed.

## 4. Discussion

This study demonstrated that the temperature response to exercise in symptomatic knee OA is affected by the different demographic and clinical characteristics of the assessed patients. Patients with a poor clinical knee status presented with a lower response to exercise, and women showed a greater temperature decrease compared to men.

The use of infrared thermography for the evaluation of musculoskeletal diseases has gaining increased interest in recent years, thanks to its simple method of evaluating the temperature of a body region, for example, for the study of tendinopathies and rheumatic diseases [4,25,26,27]. Recently, infrared thermography was proposed as a method for the evaluation of patients with knee OA to better characterize this pathology and possibly guide the treatment [4]. Although preliminary studies investigated the use of infrared thermography as a method for diagnosing and monitoring knee OA, its actual potential for use in clinical practice is still unclear, and its application remains limited [24,27,28]. In this scenario, defining how the OA knees respond to physical exercise and identifying which variables can influence this response could be useful to optimize its potential to detect OA patterns.

Previous studies evaluated the response to physical exercise in different body areas of healthy volunteers, reporting different temperature patterns [19,29,30]. In particular, significant heterogeneity among the different studies was found in terms of the response to exercise in relation to the intensity and duration of exercise [31]. Studies evaluating the skin temperature after brief exercise reported an initial temperature decrease and a subsequent temperature increase, while other studies analyzing skin temperature directly after a long bout of exercise directly detected a temperature increase compared to baseline conditions, probably hiding the initial temperature decrease [15,16]. In detail, Arfaoui et al. performed a 5-min running exercise at a speed of 8 km/h with thermalization for 30 min, a room temperature of 18 ± 0.5 °C, and a humidity of 60%, while Brito et al. performed a 50-min training session with thermalization for 10–15 min, a room temperature of 28.2 ± 0.5 °C, and a humidity of 48.1 ± 1.2%. The initial temperature decrease appeared to be due to vasoconstriction of the skin circulation and a redistribution of blood flow from the skin to the muscles involved in the exercise [29,30,32]. The following increase in temperature above the baseline values appeared to be due to the activation of cutaneous mechanisms of heat dissipation [31]. 

The current study, focusing on older patients (mean age 61 years) with knee OA, demonstrated that the temperature of symptomatic OA knees changes in response to two minutes of physical exercise with a temperature decrease immediately after exercise. This temperature change two minutes after exercise is similar to that reported in a previous study conducted by Formenti et al. [33]. Through infrared thermography, these authors analyzed the response to exercise in 13 young healthy volunteers (mean age 25 years), showing a peak temperature reduction of between two and three minutes from the beginning of exercise. However, in this study, the authors documented a subsequent increase in the temperature which was not confirmed in the different population used in the current study. The temperature of OA knees seven minutes after the beginning of exercise remained unchanged compared with that at two minutes, and it was lower than the baseline temperature. On one hand, this difference could be justified by the different participant ages between the two studies, with possible differences in the vascular response to exercise [34,35]. On the other hand, the differences could be explained by the detection of temperature in the different skin areas in the two studies. In fact, Formenti et al. analyzed the skin temperature above the quadriceps muscles, while in the current study, the temperature was evaluated above the knee joint. Interestingly, the subdivision of the knee into subareas allowed us to highlight different behaviors in different subareas following exercise. For example, the region of the patella is cooler due to the underlying bone, and other areas may respond differently. The suprapatellar area showed a response to the exercise similar to that found in the study of Formenti et al., with a temperature decrease occurring two minutes after the beginning of exercise, followed by a significant temperature increase. Perhaps this is due to the proximity of the suprapatellar area to the distal part of the quadriceps muscle, thus showing a behavior similar to that of the skin over the muscles. On the other hand, all other subareas and the total knee temperatures demonstrated an initial decrease that was not followed by a return to baseline values after this short exercise bout and at the last studied timepoint.

This study also detected a correlation between the clinical status of the patients and the thermal response of their knees to exercise. A positive correlation was found between the evaluated clinical scores and the changes in temperature after physical exercise. Patients with a better clinical status showed a greater change in temperature compared to patients with a worse clinical status. Therefore, patients with fewer symptoms demonstrated a temperature decrease comparable with that of healthy subjects, as analyzed in previous studies [30,33,36]. This trend was confirmed by subjective scores evaluating pain, such as the VAS, or more complex functional scales, such as the IKDC subjective score and the KOOS subscales. On the other hand, patients with a worse clinical status had a lower response to exercise with a reduced temperature variation. This could be explained by the fact that patients with a worse clinical status could have performed the exercise at a lower intensity, activating the muscles less. Moreover, the higher association of a worse clinical status with a higher inflammatory component in knee OA [8,37,38] could also partly explain the altered response to physical exercise. 

The response to exercise in this population also correlated with the results of the PainDETECT questionnaire, which evaluates the contribution of neuropathic pain to pain perception by the patient [39,40]. This score has not only been associated with impaired pain modulation but also with neuropathy, which may contribute to OA knee pain through damage to nerve fibers in the joint [41,42,43]. Considering that nerve fibers have a fundamental role in regulating skin circulation by releasing catecholamines, their alteration could lead to an impaired response to external stimuli, such as exercise [41,44,45]. In fact, patients in the current study with high PainDETECT questionnaire scores showed less temperature variation after exercise. This result could be explained by altered cutaneous vasoconstriction due to neuropathy, resulting in an alteration of the peripheral neuromodulation mechanisms. While the clinical relevance of this finding remains to be established, this finding confirms the presence of different factors influencing OA joints and the need to better study knee OA patterns.

The thermal response to exercise in patients with knee OA was also affected by sex. Women had a greater decrease in skin temperature than men immediately after exercise and at five minutes after its end. This could be related to differences in the metabolic, contractile, and hemodynamic properties of skeletal muscle between women and men, as well as the different cutaneous adipose tissue distribution [46,47]. Women have greater capillarization of the muscle than men and also a greater vasodilatory response of the arteries supplying the skeletal muscles, which leads to a greater increase in blood flow [48,49,50]. Moreover, women usually have a reduced exercise capacity and a lower blood volume than men; therefore, the same physical exercise could require a major effort and thus a relatively high level of blood transfer from the skin to the muscles compared to men. [51]. In previous work, it has been shown that the same type of exercise with the same number of repetitions can provide a greater training stress in women than in men [52]. In the current study, both sexes performed the same exercise for the same amount of time and at the same frequency. From this perspective, the greater activation of compensatory mechanisms aimed at redistributing the flow to the muscles involved in the exercise could explain the greater cutaneous vasoconstriction detected by the infrared thermography in women. Further studies on a larger numbers of patients should explore whether, besides the overall higher temperature changes, women present similar or different response patterns to men based on demographic, clinical, or other influencing factors.

This study presents some limitations. Although this is the largest study evaluating the thermal response to exercise in symptomatic patients with knee OA, future studies with larger populations are needed to confirm the identified correlations. Second, a control group of nonsymptomatic knee OA patients or non-OA knee patients could be used to better characterize the response of the knee to the exercise stimulus and to better evaluate temperature changes related to the presence and severity of OA disease. Third, the performed exercise may not have been optimal to generate the largest thermal response of the evaluated knee, and it could require different stresses among different patients. Therefore, future studies should investigate other possible exercises tailored to patients in terms of the type, time, and effort. It was not possible to perform evaluations using tests, such as Doppler vascular examination to exclude varicose veins or electrodiagnostic testing to better characterize the neuropathic component of the patients’ pain, and future studies should better characterize the neuropathic component of pain in these patients. Although the results obtained are statistically significant, the large interindividual variability and the many variables influencing temperature may have reduced the power of the study, so the results need future confirmation. Finally, the method of thermographic image acquisition and analysis was based on previous literature, but no method has been described as the gold standard in this field. For example, we adopted a 10-min protocol for patient thermalization, while other authors prefer a 15-min window of thermalization before the thermographic evaluation. It is possible that different settings, different lenses, and different devices could be more suitable for such evaluations in clinical practice. The standardization of thermography use for the evaluation of knee OA could improve its potential for identifying different disease patterns both in research and in clinical practice. In this regard, this study provides new input on how the thermographic findings can be influenced by simple exercise testing, which could be useful for studying patients and knees with different OA patterns so that they can be targeted by specific and more effective treatment approaches in the future.

## 5. Conclusions

This study demonstrated that the temperature response to knee flexion–extension exercise in symptomatic knee OA is affected by the demographic and clinical characteristics of the assessed patients. Patients with a poor clinical knee status presented a lower response to exercise, and women showed a greater temperature decrease compared to men. Not all evaluated ROIs showed the same trend, which underlines the need to specifically study the different joint subareas to identify the inflammatory component and joint response while investigating knee OA patterns.

## Figures and Tables

**Table 1:** Included patients’ characteristics.

**Sex, M/W**	38/22
**Age, years**	61.4 ± 9.2 (43–75)
**BMI, kg/m^2^**	25.4 ± 3.0 (19.5–33.8)
**Side**	Right: 33—Left: 27
**Symptom duration, months**	108.3 ± 99.3 (18–372)
**Symptom onset**	Acute: 14—Chronic: 46
**Previous knee surgery, yes/no**	31/29
**Smoker, yes/no**	13/47
**Kellgren–Lawrence grade**	Grade 2: 30
	Grade 3: 21
	Grade 4: 9
**VAS pain**	5.6 ± 2.3 (1–9)
**IKDC subjective score**	41.3 ± 14.2 (9.2–81.6)
**IKDC objective score**	Grade 1: 8
	Grade 2: 29
	Grade 3: 10
	Grade 4: 13
**KOOS pain**	59.8 ± 18.9 (2–94)
**KOOS symptoms**	60.4 ± 19.7 (18–100)
**KOOS ADL**	69.5 ± 18.4 (6–100)
**KOOS QoL**	34.5 ± 16.2 (0–75)
**KOOS Sport/Rec**	43.8 ± 17.7 (20–90)
**Tegner score pre-treatment**	2.2 ± 1.2 (1–5)
**PainDETECT questionnaire**	8.7 ± 5.5 (0–25)

Values are expressed as mean ± standard deviation and range (). ADL, Activities of daily living; BMI, body mass index; IKDC, International Knee Documentation Committee; KOOS, Knee Injury and Osteoarthritis Outcome Score; M, men; QoL, Quality of Life; Sport/Rec, Function in Sport and Recreation; VAS, visual analogue scale; W, women.

## Data Availability

Not applicable.
